# Targeting inflammation in perivascular cells and neuroimmune interactions for treating kidney disease

**DOI:** 10.1007/s10157-024-02494-7

**Published:** 2024-04-17

**Authors:** Shinji Tanaka

**Affiliations:** Division of Nephrology and Endocrinology, The University of Tokyo Graduate School of Medicine, 7-3-1 Hongo, Bunkyo-Ku, Tokyo, 113-8655 Japan

**Keywords:** Fibroblasts, Pericytes, Neuroimmune interactions, Vagus nerve stimulation, Acute kidney injury, Chronic kidney disease

## Abstract

Inflammation plays a crucial role in the pathophysiology of various kidney diseases. Kidney perivascular cells (pericytes/fibroblasts) are responsible for producing proinflammatory molecules, promoting immune cell infiltration, and enhancing inflammation. Vascular adhesion protein-1, expressed in kidney perivascular cells, is an ectoenzyme that catalyzes the oxidative deamination of primary amines with the production of hydrogen peroxide in the extracellular space. Our study demonstrated that blocking this enzyme suppressed hydrogen peroxide production and neutrophil infiltration, thereby reducing renal ischemia–reperfusion injury. Sphingosine 1-phosphate (S1P) signaling was also observed to play an essential role in the regulation of perivascular inflammation. S1P, which is produced in kidney perivascular cells, is transported into the extracellular space via spinster homolog 2, and then binds to S1P receptor-1 expressed in perivascular cells. Upon injury, inflammatory signaling in perivascular cells is enhanced by this pathway, thereby promoting immune cell infiltration and subsequent fibrosis. Furthermore, inhibition of S1P transport by spinster homolog 2 reduces kidney fibrosis. Hypoxia-inducible factor-prolyl hydroxylase inhibitors can restore the capacity for erythropoietin production in kidney perivascular cells. Animal data suggested that these drugs could also alleviate kidney and lipid inflammation although the precise mechanism is still unknown. Neuroimmune interactions have been attracting significant attention due to their potential to benefit patients with inflammatory diseases. Vagus nerve stimulation is one of the most promising strategies for harnessing neuroimmune interactions and attenuating inflammation associated with various diseases, including kidney disease. Using cutting-edge tools, the vagal afferents–C1 neurons–sympathetic nervous system–splenic nerve–spleen–kidney axis responsible for kidney protection induced by vagus nerve stimulation was identified in our study. Further research is required to decipher other crucial systems that control kidney inflammation and to determine whether these novel strategies can be applied to patients with kidney disease.

## Introduction

Acute kidney injury (AKI), which is characterized by a rapid loss of kidney function, affects approximately 10–15% of patients admitted in hospitals, and in intensive care units, its incidence can be as high as 50% [1]. Inflammation plays a critical role in the pathogenesis of AKI [2]. AKI is associated with high morbidity and mortality [3]. Moreover, recent epidemiological and experimental observations have also indicated that AKI can progress to chronic kidney disease (CKD) [4, 5]. Approximately 10% of the population worldwide suffers from CKD, which is associated with sustained inflammation and progressive fibrosis in the kidney [6, 7]. CKD can progress to end-stage kidney disease and is a potential risk factor for cardiovascular disease [8, 9]. Currently, the treatment options available to alleviate AKI are quite limited [1], and even with widespread use of renin–angiotensin system blockers and sodium–glucose cotransporter 2 inhibitors, a substantial residual risk of CKD progression remains [10, 11]. Hence, it is important to understand the role of inflammation in the pathogenesis of AKI/CKD as it can lead to novel therapeutic strategies. In this review, we discuss recent advances in understanding the role of inflammation in kidney disease with potential therapeutic strategies.

## Perivascular cells play a crucial role in kidney inflammation

Innate immunity is critical to various types of AKI (e.g., ischemia) [12]. The first step in the infiltration of immune cells into injured kidneys is the interaction between immune cells and adhesion molecules, such as selections, intercellular adhesion molecule-1, and vascular cell adhesion molecule-1, expressed in endothelial cells. Blocking this step successfully ameliorated AKI in animal studies [13, 14]; however, translation of this therapeutic concept into clinical settings has not been very successful [15]. Thus, an alternative strategy to block immune cell infiltration is required for the treatment of AKI.

**Fig. 1:**
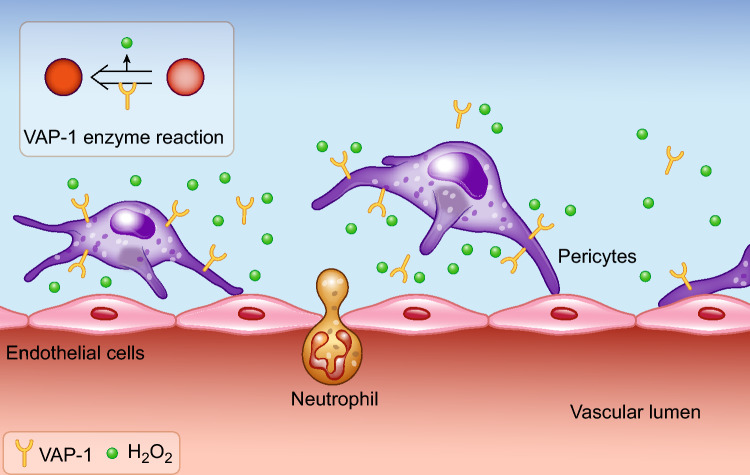
VAP-1 in pericytes enhances neutrophil infiltration into the injured kidney by generating hydrogen peroxide (H_2_O_2_). Reprinted from [20], with permission from International Society of Nephrology (Elsevier). VAP-1 expressed in pericytes catalyzes the oxidative deamination of primary amines, resulting in the production of H_2_O_2_ in the extracellular space. This generates a local H_2_O_2_ gradient, which in turn enhances the infiltration of neutrophils into the injured kidney, thus worsening the injury

An ectoenzyme, vascular adhesion protein-1 (VAP-1), is a 170–180 kDa homodimeric transmembrane glycoprotein that catalyzes the oxidative deamination of primary amines in the extracellular space (R-CH_2_-NH_2_ + O_2_ + H_2_O → R-CHO + NH_3_ + H_2_O_2_) [16, 17]. This molecule is known to be important for immune cell infiltration [18, 19], but its mechanism remains unknown. Several researchers along with ourselves, have observed the predominant expression of VAP-1 in perivascular cells (pericytes/fibroblasts) in the kidney and the liver [20, 21]. Pharmacological inhibition of VAP-1 has been observed to reduce rat kidney ischemia/reperfusion injury (IRI) through the suppression of neutrophil infiltration [20]. Hydrogen peroxide is a critical chemoattractant for neutrophils [22, 23], and our study demonstrated that hydrogen peroxide, a product generated by the VAP-1 enzyme reaction, aggravated AKI by attracting neutrophils into injured kidneys (Fig. 1).

**Fig. 2:**
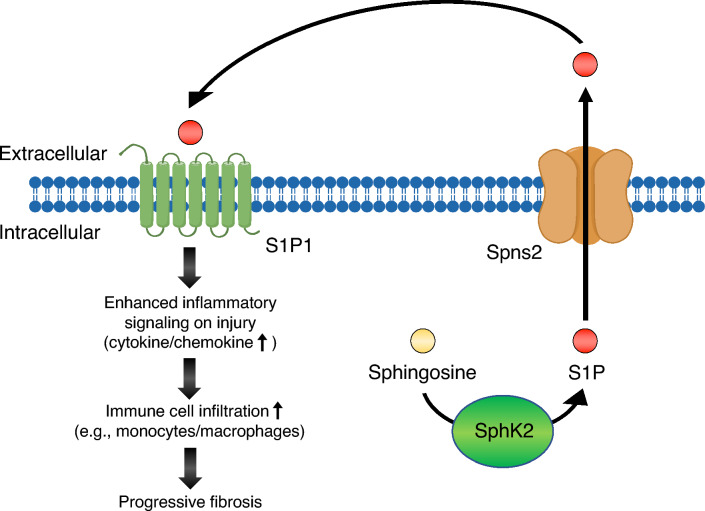
SphK2/S1P/Spns2/S1P1 axis enhances inflammatory signaling in kidney perivascular cells (reprinted from [27]). S1P, which is predominantly produced by SphK2 in kidney perivascular cells, is transported into the extracellular space through Spns2 and binds to S1P1 in an autocrine manner. This axis enhances inflammatory signaling through production of proinflammatory cytokines/chemokines on injury, which promotes immune cell infiltration and subsequent fibrosis

Perivascular cells are also known to contribute to the progression of fibrosis by attracting and navigating immune cells to inflammatory sites [20, 24]. Kidney perivascular cells function as major innate immune sentinels and express adhesion molecules and proinflammatory cytokines/chemokines upon injury, thereby enhancing immune cell infiltration [24, 25], which results in persistent inflammation and subsequent fibrosis [26]. A recent study conducted by us has demonstrated that sphingosine 1-phosphate (S1P) signaling enhances perivascular inflammation [27]. S1P, which is a product of sphingolipid catabolism, mediates several fundamental cellular functions, such as adhesion, proliferation, and inflammation. S1P is produced by sphingosine kinase 2 (SphK2) in kidney perivascular cells, and exported through spinster homolog 2 (Spns2) into the extracellular space. Extracellular S1P then binds to the S1P receptor-1 (S1P1) that is expressed in perivascular cells [27]. The SphK2/S1P/Spns2/S1P1 axis enhances perivascular inflammatory signaling, promoting immune cell infiltration and subsequent fibrosis in the event of kidney injury. A small-molecule Spns2 inhibitor [28], which had been previously developed by us, successfully suppressed inflammatory signaling in kidney perivascular cells and ameliorated kidney fibrosis (Fig. 2). The significance of perivascular inflammatory signaling has been demonstrated in other organs as well. Following injury, pericytes in the lung produce proinflammatory cytokines/chemokines [29], and an inflammatory response in liver pericytes also contributes to inflammation and fibrosis [30, 31]. Hence, perivascular inflammation could serve as a good drug target for the treatment of AKI and CKD.

Kidney perivascular cells perform the additional function of producing erythropoietin (EPO) in response to hypoxia, which binds to erythroid progenitors in the bone marrow and enhances erythropoiesis [32, 33]. In the event of kidney injury, EPO-producing perivascular cells lose their capacity to produce EPO by transdifferentiating into myofibroblasts [32–35]. This describes the pathophysiology of anemia in CKD. Hypoxia-inducible factor-prolyl hydroxylase inhibitors (HIF-PHIs) can restore the capacity for EPO production [32–34]. Hypoxia-inducible factor (HIF) and HIF-prolyl hydroxylase (HIF-PH) play major roles in regulating EPO production in kidney perivascular cells [36, 37]. HIF, which consists of an oxygen-sensitive α subunit (HIF-α) and a constitutively expressed β subunit (HIF-β), is a heterodimeric transcription factor. HIF-α is consistently synthesized in cells; however, under normoxic conditions, hydroxylation by HIF-PH at specific proline residues enables recognition of HIF-α by the von Hippel–Lindau–E3 ubiquitin ligase complex, resulting in its degradation. Conversely, hypoxic conditions lead to HIF-α accumulation since the hydroxylation of HIF-α does not occur due to scarcity of oxygen. Then, HIF-α and HIF-β bind to the hypoxia-response element, thereby inducing EPO transcription. In addition to the regulation of EPO transcription, this system induces a broad spectrum of genes important for adaptation to hypoxia in virtually all mammalian cells. Three isoforms of HIF-α (HIF-1α, HIF-2α, and HIF-3α) and HIF-PH enzymes (HIF-PH1, HIF-PH2, and HIF-PH3) have been identified, among which the HIF-PH2/HIF-2α axis plays a predominant role in the regulation of EPO production in the kidney [38, 39]. Since HIF-PH enzymes are known to regulate a broad spectrum of genes, and given that HIF-PHIs can affect cells other than EPO-producing kidney perivascular cells, in addition to the effect on EPO induction, HIF-PHIs can have off-target effects, both favorable and unfavorable. Previous exploration of the long-term effect of HIF-PH inhibition in diabetic mice by us [40] demonstrated that the administration of HIF-PHI caused suppressed macrophage infiltration in glomeruli and white adipose tissue, which was accompanied by improved glucose and lipid metabolism, reduced albuminuria, and suppressed epithelial/endothelial damage in the glomeruli. The mechanism by which HIF-PHI suppressed inflammation in glomeruli and white adipose tissue remains unknown; however, in vitro studies have indicated that HIF-1 activation resulted in the suppression of MCP-1 production in mesangial cells. To date, there are no clinical data to directly show that HIF-PHIs suppress inflammation or kidney injury. This discrepancy might be due to, at least in part, the difference between CKD patients with anemia and animal models (e.g., kidney function). Further preclinical and clinical studies investigating the effects of HIF-PHIs on the kidney and whole body are necessary [41, 42].

Perivascular cells play several harmful and beneficial roles (e.g., promoting tubular regeneration) in the kidney. Please refer to a recent review [43] that describes the multiple functions of kidney perivascular cells in physiological and pathophysiological states.

## Utilization of neuroimmune interactions to alleviate kidney inflammation

Pharmacological approaches to alleviate kidney inflammation have not met with much success in clinical trials, partly because drug targets are often embedded within pathways that exhibit high redundancy. Excessive immunosuppression by drugs must be avoided in patients with kidney disease because they already have a greater possibility of developing infectious diseases. Thus, nonpharmacological approaches to alleviate kidney inflammation are more eagerly awaited. Among these approaches, the use of neuroimmune interactions is gathering more attention because studies have revealed neural pathways that regulate inflammation [44, 45].

In 1995, Watkins et al. found that subdiaphragmatic vagal transection abolished IL-1β-induced hyperthermia, indicating the activation of vagal afferents caused by peripheral inflammation, to initiate fever response [46]. At the beginning of the 2000s, Kevin Tracey and colleagues demonstrated that a small amount of CNI-1493 (potent antiinflammatory agent) administered via the intracerebroventricular route decreased tumor necrosis factor (TNF), not only in the brain but also in the plasma, originating predominantly from the spleen, in rats treated with LPS [47]. They also found that cutting the vagus nerve annulled the decrease in plasma TNF caused by the treatment with CNI-1493 and that electrical stimulation of the vagus nerve was sufficient to reduce plasma TNF, indicating that some signals alleviate inflammation by descending from the brain through the vagus nerve to the spleen. “The inflammatory reflex” was advocated based on these findings; after the afferent vagus nerve senses inflammation occurring in the periphery, the signal is transmitted to the efferent vagus nerve to abrogate the peripheral inflammation.

**Fig. 3:**
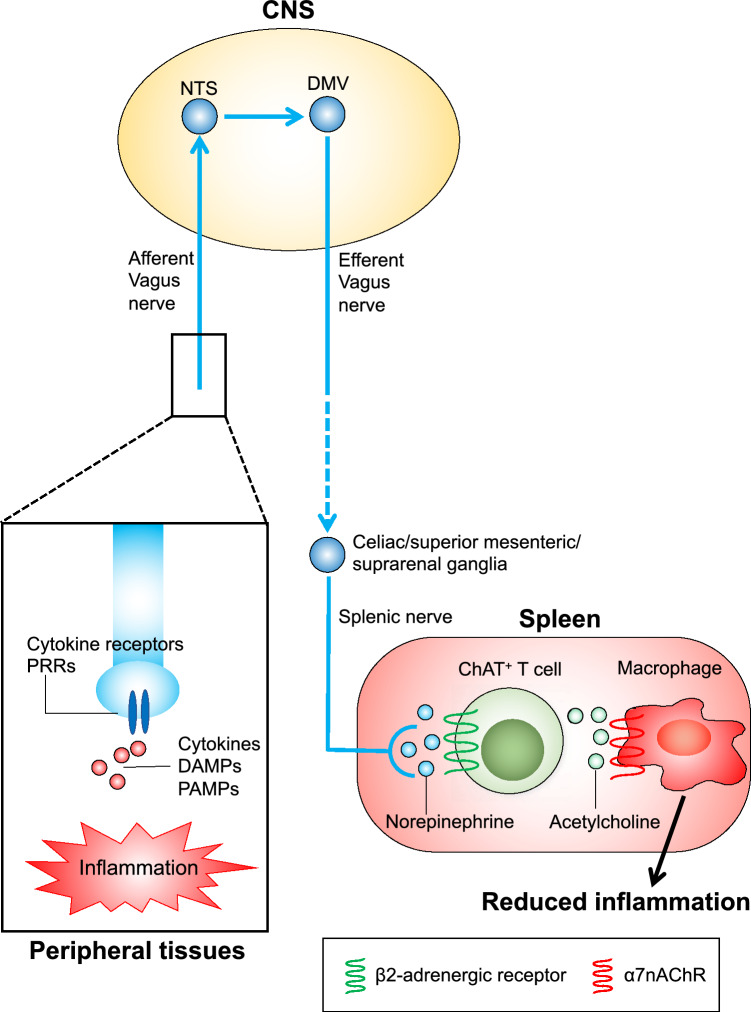
The inflammatory reflex. Reprinted from [44], with permission from Elsevier. Please refer to the text for details. *DAMPs* damage-associated molecular patterns, *PAMPs* pathogen-associated molecular patterns, *PRRs* pattern recognition receptors, *CNS* central nervous system, *ChAT* choline acetyltransferase, *DMV* dorsal motor nucleus of the vagus, *NTS* nucleus tractus solitarius, *α7nAChR* α7 nicotinic acetylcholine receptor

Extensive efforts have been made to understand the mechanism of the inflammatory reflex. The current concept of the inflammatory reflex is as follows (Fig. 3): (1) In case of peripheral inflammation, afferent vagus nerve terminals are stimulated by inflammatory products, that include proinflammatory cytokines, damage-associated molecular patterns, and pathogen-associated molecular patterns, through cytokine receptors and pattern recognition receptors expressed on vagal afferents [48]. (2) Transmission of nerve activity in the brain causes activation of the efferent vagus nerve. (3) The signal is relayed to the splenic nerve (primarily sympathetic) [49]. (4) The splenic nerve terminals release norepinephrine, which binds to β_2_-adrenergic receptors expressed on choline acetyltransferase (ChAT)-positive T cells in the spleen. This causes release of acetylcholine (ACh) from this specific T cell subpopulation [50]. (5) The interaction of ACh with α7 nicotinic ACh receptors (α7nAChRs) expressed on macrophages residing close to ChAT-positive T cells, suppresses proinflammatory cytokine production and inflammation [51]. The efferent arm of the inflammatory reflex is referred to as the cholinergic antiinflammatory pathway (CAP) [52].

**Fig. 4:**
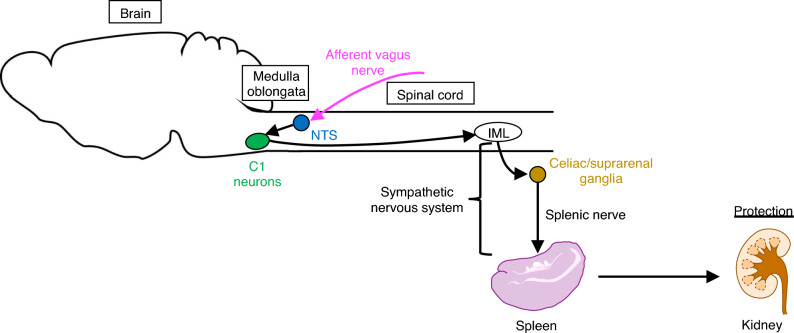
Proposed mechanism of kidney protection by vagal afferent stimulation (reprinted from [55]). Please refer to the text for details. *NTS* nucleus tractus solitaries, *IML* intermediolateral cell column

Considering the dominant role played by the vagus nerve in the inflammatory reflex, vagus nerve stimulation (VNS) can be considered as a reasonable therapeutic strategy for inflammatory diseases. Inoue et al. demonstrated that VNS performed 24 h before kidney IRI protected the kidney [53]. VNS performed in brain dead donor rats also improved long-term kidney function and survival of recipients by suppressing immune cell infiltration into tubules and arteries [54]. Electrical stimulation of the vagus nerve causes transmission of action potential in both types of fibers (efferent motor and afferent sensory fibers). Optogenetics was recently utilized to elucidate the precise neural circuits involved in kidney protection induced by VNS [55]. Optogenetics is a technique that optically controls cells in living tissues, typically neurons, that have been genetically modified to express light-sensitive opsins. Channelrhodopsin-2 (ChR2), a representative light-sensitive excitatory opsin, is a nonselective cation channel, and its gate rapidly opens following application of blue light. Thus, ChR2-expressing neurons can be selectively depolarized by blue light, mainly via Na^+^ entry, thereby evoking an action potential. *Chat*-*ChR2* and *Vglut2*-*ChR2* mice, in which ChR2 is expressed in the efferent and afferent vagus nerve respectively, were used in our experiments. Through the experiments with optogenetic selective VNS, either efferent or afferent fiber stimulation was demonstrated to be sufficient to protect the kidneys against IRI. We further identified a new pathway, the C1 neurons (in the medulla oblongata)–sympathetic nervous system–splenic nerve–spleen–kidney axis that protects the kidney through vagal afferent stimulation (Fig. 4) [55]. This significant role of C1 neurons in kidney protection is in agreement to a study conducted by Abe et al., in which they demonstrated that restraint stress was protective against kidney IRI through the activation of C1 neurons and that optogenetic stimulation of C1 neurons was also sufficient for kidney protection [56]. Adoptive transfer of splenocytes, but not lymph node/bone marrow cells, isolated from VNS-treated mice protected naïve recipient mice from kidney IRI, further supporting a critical role of splenocytes in this context [55]. These results indicate that the signal from the splenic nerve protects the kidney by altering the phenotype of the splenocytes; however, its mechanism needs to be elucidated.

VNS is an effective therapy that has already received FDA approval for the treatment of various disorders, such as epilepsy, depression, migraines, cluster headaches, and obesity [57]. Several clinical trials have been performed and are ongoing to convert the protective effect of VNS into treatment for inflammatory diseases. The first clinical trial that tested an implanted electronic device in patients suffering from refractory rheumatoid arthritis demonstrated inhibition of TNF by VNS, which resulted in improved disease severity for up to 84 days [58]. The effectiveness of VNS has also been tested in Crohn’s disease [59]. VNS enabled achievement of significant clinical remission (decreased disease activity index and improved endoscopic findings) for 6 months in five out of seven patients with active disease. These results demonstrate that VNS can also alleviate inflammation in humans, offering a promising treatment option of using VNS for treating other inflammatory diseases, including kidney disease. Larger clinical trials and continued investigations elucidating the mechanism by which VNS exerts an antiinflammatory effect are warranted for the safe and effective clinical application of VNS in patients with kidney disease.

## Conclusion

Inflammation is evidently a critical factor in the pathophysiology of several kidney diseases. Accumulating data indicates that kidney perivascular cells use inflammatory signaling to attract immune cells to the injury site, thereby enhancing inflammation. Thus, perivascular cells could serve as a reasonable drug target for treating kidney disease. Another strategy is utilization of neuroimmune interaction, which can be a nonpharmacological approach. VNS is one of the most promising approaches for harnessing neuroimmune interactions and attenuating inflammation. Further studies are required to precisely determine the mechanisms by which these strategies exert antiinflammatory effects and to further apply them in clinical settings.
